# Assessment of dental ontogeny in late Miocene hipparionines from the Lamagou fauna of Fugu, Shaanxi Province, China

**DOI:** 10.1371/journal.pone.0175460

**Published:** 2017-04-26

**Authors:** Yangfan Li, Tao Deng, Hong Hua, Yongxiang Li, Yunxiang Zhang

**Affiliations:** 1 Institute of Cenozoic Geology and Environment, State Key Laboratory of Continental Dynamics, and Department of Geology, Northwest University, Xi’an, China; 2 Key Laboratory of Vertebrate Evolution and Human Origins, Institute of Vertebrate Paleontology and Paleoanthropology, Chinese Academy of Sciences, Beijing, China; 3 CAS Center for Excellence in Tibetan Plateau Earth Sciences, Beijing, China; 4 University of Chinese Academy of Sciences, Beijing, China; New York Institute of Technology, UNITED STATES

## Abstract

A collection of 28 hipparionine skull and mandible fossils with a dated age of approximately 7.4 Ma from Fugu, Shaanxi, northwestern China (belonging to *Hipparion chiai* and *Hipparion* cf. *coelophyes*) shows an age distribution in a successive development sequence. By observing the dentitions in these fossil materials, knowledge of dental ontogeny has been gained, such as the opening time of the posterior wall of post-fossettes, the displacement of the plis hypostyle, the morphologic changes of the protocone and hypocone, etc. Additionally, 4 isolated maxillary cheek teeth and 2 mandibular cheek teeth were cut into slices in the traditional manner for authentication. These discoveries indicate that both of the hipparionine species in the Lamagou fauna are *Hipparion* cf. *chiai* exactly and offer further insight into the morphologic changes that occur during dental wear in hipparionines, which may greatly promote the morphological and taxonomic study of hipparionine species.

## Introduction

Hipparionine horse has significant value on evolutionary biology, stratigraphy, and palaeoecology as the index fossil in the Cenozoic strata. A key problem in taxonomy of hipparionine horses is the high intraspecific variations and the less obvious species differences, and the occlusal structural change with dental wear is one of the great causes of these.

In the last 100 years, special attention has been focused on the dental ontogeny in hipparionine horses, such as in the work of Gidley [1], Teryaev [2], Wehrli [3], Gromova [4], Qiu et al. [5], Pang [6] and Bernor and Sun [7]. The most common method is to characterize and analyze the cheek teeth sections to simulate the ontogenesis, but the variations associated with teeth growth might be neglected in this idealized examination. Death not only marks the end of life but also creates a snapshot of the growth stage. Therefore, fossils are good carriers of information on growth stages. Using a set of the same fossils, a parts or even a complete ontogenetic sequence can be created. Indeed, this method has been widely used in ontogeny and morphology, e.g., auxology has been one of the main research fields in Trilobite development [8–10]. A complete sequence of skull fossils of *Platybelodon* from the Linxia Basin is highly surprising because it displays the entire ontogenesis.

## Methods and materials

### Abbreviation

NWU: vertebrate fossil number of the Department of Geology, Northwest University, Xi’an, China.

### Measurements

Measurement numbers on maxillary and mandibular cheek tooth (M1, M2, M3, etc.) conform to the standards published by Eisenmann et al. [11] for the “Studying Fossil Horses”. In addition, for the sectioned teeth reported herein, the first column is replaced by providing the composite crown height of the sectioned cheek tooth; otherwise, the numbers of plications on the posterior wall of the prefossette in the plication formula is divided into: “pli (s) protoconule + pli (s) prefossette”. The measurement results were shown in S1–S6 Tables.

### Materials

**Fig 1:**
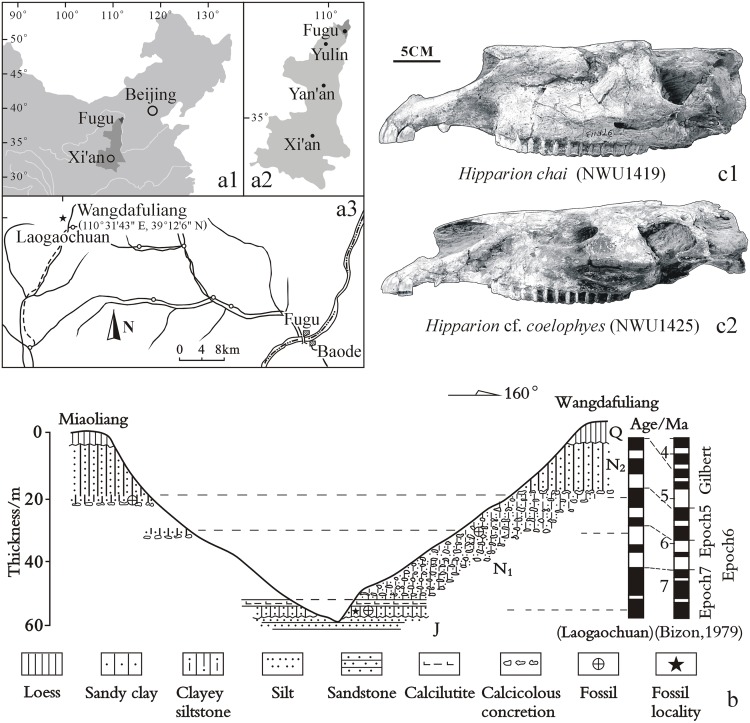
Location and section of Wangdafuliang in Fugu, Shaanxi, China, and the hipparionine skulls from the Lamagou fauna. (A) North China with the location of Shaanxi; (B) Fugu with the location of Shaanxi; (C) Wangdafuliang with the location of Fugu; (D) Cross-section of Wangdafuliang [10]; (E) skull of *Hipparion chiai* from the Lamagou fauna; (F) skull of *H*. cf. *coelophyes* from the Lamagou fauna.

The hipparionine fossils were recovered in Lamagou fauna from Fugu, Shaanxi (Fig 1A, 1B and 1C): (1) 11 skulls and 17 mandibles were collected from the lower fossil bed (with a paleomagnetic age of approximately 7.4 Ma) at the Wangdafuliang Section (comprising 3 fossil beds in this section, Fig 1D) [12, 13]. (2) These 27 fossils (NWU1426, including the skull and lower jaw) were approximately identified as *Hipparion chiai* (Fig 1E) and *H*. cf. *coelophyes* (Fig 1F) [12–14], and the morphological features of these two species are closely related. (3) The 27 fossils originate from different ages; therefore, they form a relatively complete ontogenetic sequence and are highly suitable for the assessment of dental ontogeny. For comparison, 4 isolated and less-worn hipparionine maxillary cheek teeth and 2 mandibular cheek teeth from Lamagou fauna were cut into slices with the traditional method for use as an auxiliary group.

### Methods

The ontogenetic sequences in ascending age order are listed as follows: (1) Fossils with a typical age mark are selected as standard references, such as juveniles in the teething stage and adults with relatively complete incisors; (2) By comparing the known modes of tooth wear with the standard references (such as the occlusal length, the cuspidal breadth, etc.), the age of other samples can be estimated. Judgments on and age grades are referenced to “The Chinese Hipparionine Fossils” [5]. The eruption sequence follows “The Anatomy of the Domestic Animals” [15], and the eruption ages are converted in equal proportions of 3.5 years:

DI/di1, DI/di2-DI/di4 New born-Half month

DI/di2 Half month-One month

P/p1 or DP/dp1 4 to 5 months

DI/di3 5 to 7 months

M/m1 7 to 10 months

M/m2 ~1.5 years

I/i1, P/p2 ~2 years

P/p3 2 to 2.5 years

I/i2 2.5 to 3 years

P/p4, M/m3 ~3 years

I/i3 ~3.5 years

C/c 3 to 3.5 years

Young adulthood (Early wear) 3.5 to 8 years

Middle adulthood (Middle wear) 8 to 12 years

Senility (Late wear) 12 to15 years

**Fig 2:**
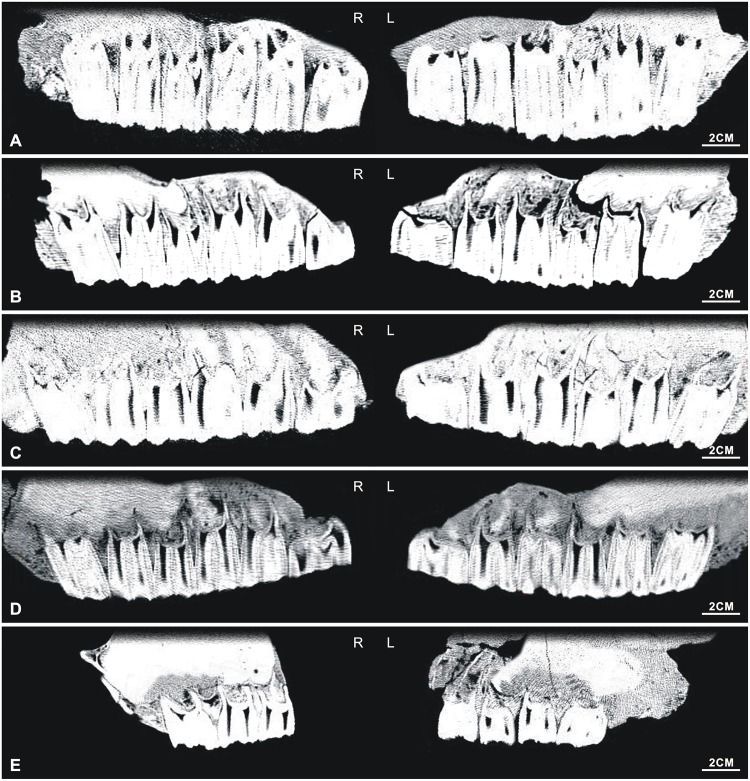
CT reconstruction images of the inlaid maxillary cheek teeth. (A) NWU1420; (B) NWU1421; (C) NWU1419; (D) NWU1425; (E) NWU1423.

**Fig 3:**
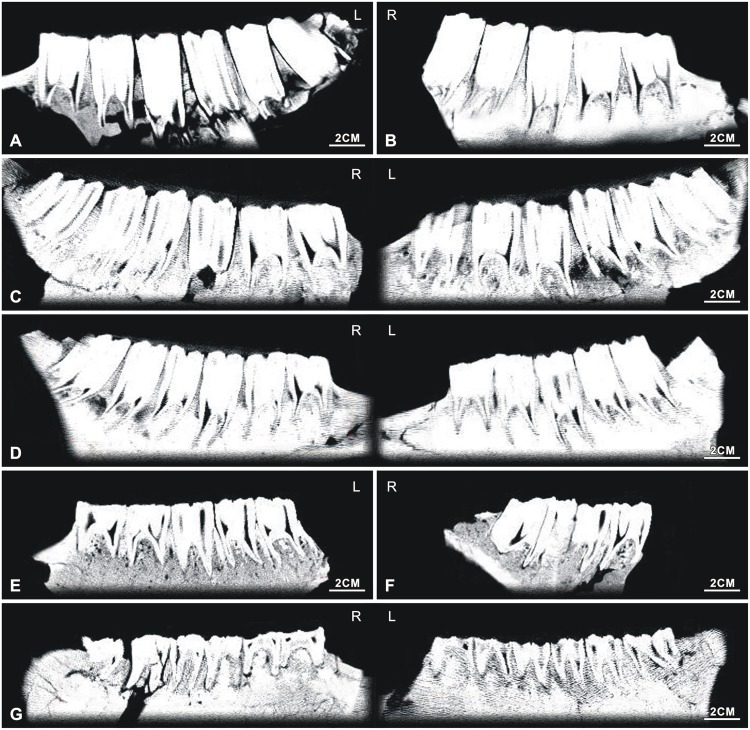
CT reconstruction images of the inlaid mandibular cheek teeth. (A) NWU1440.1; (B) NWU1439; (C) NWU1433; (D) NWU1430; (E) NWU1436; (F) NWU1444; (G) NWU1445.

Finally, to confirm the sorting’s effectiveness, the tooth height of adults in the ontogenetic sequences was measured. The inlaid teeth were viewed and measured with spiral computed tomography (CT), on a Siemens SOMATOM Emotion 16 (as shown in Figs 2 and 3, S1–S4 Tables).

The tooth height of unworn or little worn P3/p3-P4/p4 and M1/m1-M2/m2 is about 52 mm, and the hypsodonty index (HI) is 4.4. The observations were segregated into 3 parts: 1. Early wear—first 55% of tooth wear; Middle wear—second 35% of wear; Late wear—last 20% of wear. As the highlight, Early wear was further divided into 5 five stages: Early wear I (age 3.5 to 4); Early wear II (age 4 to 5); Early wear III (age 5 to 6); Early wear IV (age 6 to 7); Early wear V (age 7 to 8). Considering the factors such as eruption sequence, single tooth height difference and different wear rate, the ranks based on individual average tooth height, the single tooth height as assistance. Therefore, it seems to be deviating toward the older part of the spectrum in comparison with the traditional viewpoint. The ranks are entirely consistent with the age character of incisors in the ontogenetic sequences.

## Ontogenetic sequences and analysis

### Ontogenetic sequences of the maxillary dentitions

The ontogenetic sequences of hipparionine maxillary dentitions from Lamagou fauna are listed as follows. The samples dotted with “*” are standard references of ages. The genders of the samples without labels are unknown:

#### Ontogenetic sequences of the maxillary dentitions of *Hipparionchiai*

**Fig 4:**
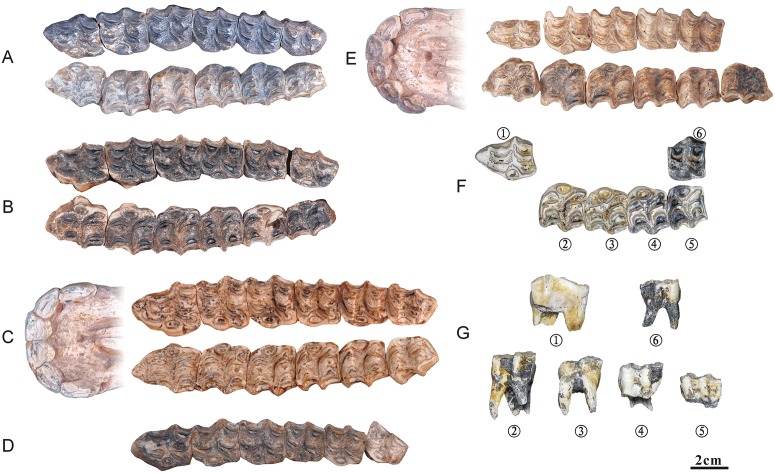
Ontogenetic sequences of the maxillary dentitions of *Hipparion chiai*. (A) NWU1420; (B) NWU1421; (C) NWU1419; (D) NWU1429; (E) NWU1424; (F) NWU1475.1- NWU1475.6; (G) the lateral view of NWU1475.1- NWU1475.6.

*NWU1420 (Figs 2A and 4A), Early wear I (age 3.5 to 4), the average tooth height is 44.3 mm;
NWU1421 (Figs 2B and 4B), male, Early wear III (age 5 to 6), the average tooth height is 33.4 mm;
*NWU1419 (Figs 2C and 4C), Early wear IV (age 6 to 7), the average tooth height is 31.3 mm;
NWU1429 (Fig 4D), Early wear V (age 7 to 8), the M3’s tooth height is 32.8 mm;
*NWU1424 (Fig 4E), female, Middle wear (approximately 9 years), the M3’s tooth height is 24.8 mm;
*NWU1475.1-NWU1475.6 (Fig 4F and 4G), 5 isolated maxillary cheek teeth from the same apparent individual, Late wear(over 12 years old), the average tooth height is 13.7 mm.
  The measurements are shown in S1 Table.

#### Ontogenetic sequences of the maxillary dentitions of *Hipparion* cf. *coelophyes*

**Fig 5:**
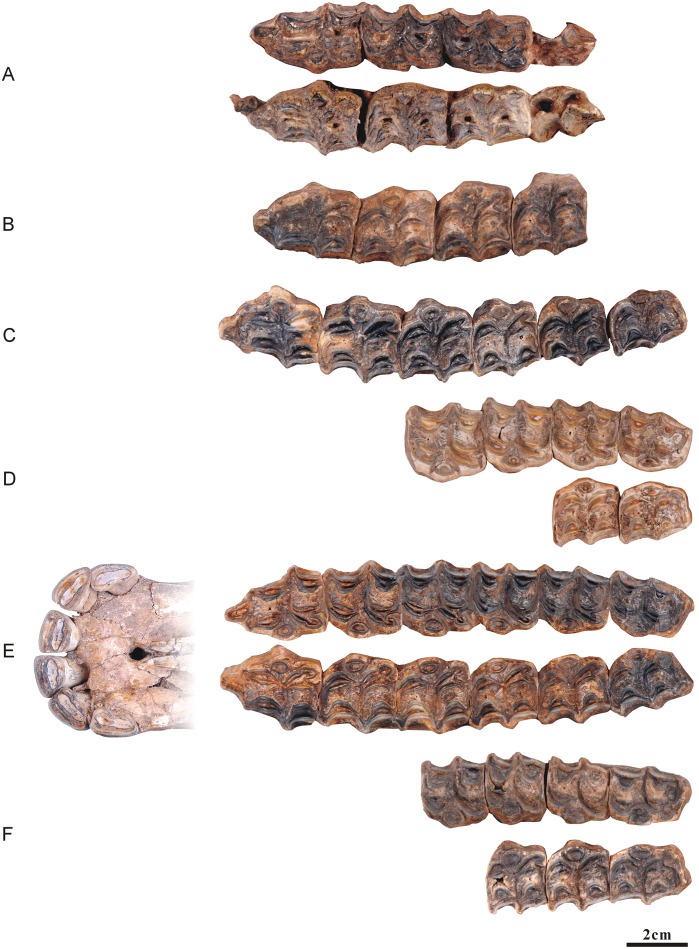
Ontogenetic sequences of the maxillary dentitions of *Hipparion* cf. *coelophyes*. (A) NWU1426.1; (B) NWU1422; (C) NWU1428; (D) NWU1427; (E) NWU1425; (F) NWU1423.

*NWU1426.1 (Fig 5A), female, juvenile phase (approximately 3/4 year);
NWU1422 (Fig 5B), male, Early wear II (age 4 to 5), the average tooth height is 34.7 mm (right M2’s tooth height is about 44.3 mm presumably by the tooth socket);
NWU1428 (Fig 5C), Early wear II (approximately 5 years), the M3’s tooth height is 39.4 mm;
NWU1427 (Fig 5D), Early wear IV (approximately 7 years), the average tooth height for the P4, M2 and M3 is 13.7 mm;
*NWU1425 (Figs 2D and 5E), female, Early wear V (age 7 to 8), the average tooth height is 28.8 mm.
NWU1423 (Figs 2E and 4E), Middle wear (age 9 to 10), the average tooth height is 20.2mm.
  The measurements are shown in S2 Table.

#### Analysis of the maxillary dentitions sequences

**Fig 6:**
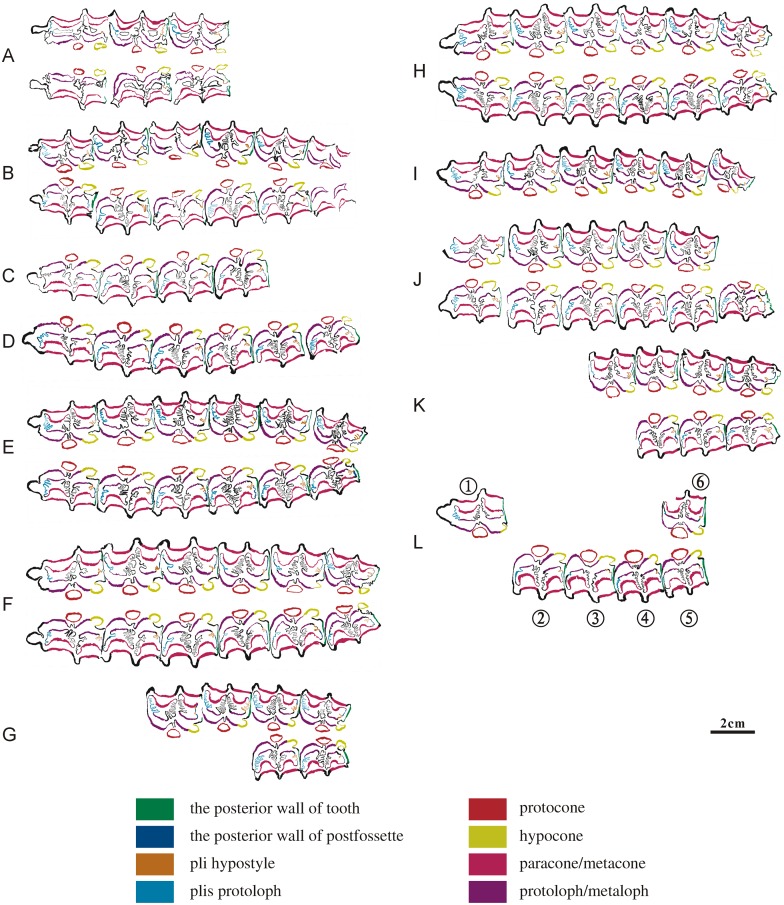
Ontogenetic sequences of hipparionine maxillary dentitions from Lamagou fauna (color line diagram). (A) NWU1426.1; (B) NWU1420; (C) NWU1422; (D) NWU1428; (E) NWU1421; (F) NWU1419; (G) NWU1427; (H) NWU1425; (I) NWU1429; (J) NWU1424; (K) NWU1423; (L) NWU1475.1- NWU1475.6.

The morphological characteristics of *Hipparion chiai* and *H*. cf. *coelophys* are highly similar. Certain small differentiations did exist in the two species, but the variation trends in tooth wear are highly consistent. Therefore, the age variation of the tooth wear can be analyzed uniformly (Fig 6):

1. In our observation, the opening of the posterior wall of postfossette is a temporary phenomenon in Early wear III to V (age 5 to 8) from P2 to P3 of our samples, and it reclosed later on. The posterior wall of the postfossette closes up the tooth posterior wall after reclosing.
2. The location of the pli hypostyle was obviously changed during the initial stage (the connecting process of the metastyle and the hypostyle and also the formation process of the closed postfossette). The pli hypostyle was located in the lingual wall of the postfossette and pointed to the labial before the postfossette closed, which can be observed clearly from the premolars of NWU1420 (Fig 6B) and is also proven in the following step sections. When the postfossette closed completely, the location of the pli hypostyle was moved to the posteromedial corner of the postfossette and remained relatively steady. It is worth noting that the pli hypostyle is located more often on the posterior wall of the postfossette than in the molars. The plications in the fossas are gradually shortened from Middle wear (after 8 years).
3. The protocone changes are described as follows. In Early wear I (age 3.5 to 4), most protocones in a dentition (P2, P4, M2 and M3) are small and elongated with a length/width ratio of approximately 2/1 and generally have a flattened lingual surface and are rounded labially, with both anterior and posterior ends pointed. In Early wear II (age 4 to 5), the protocones broaden and both the anterior and posterior ends of the premolars become rounded. In Early wear III (age 5 to 6), the protocones elongate again. In Early wear IV (age 6 to 7) as the previous stage. In Early wear V (age 7 to 8), the protocones are shorter and very similar with Early wear II. From Middle wear to Late wear (after 8 years), the protocones become wildly ovate from anterior to posterior in a gradual manner. The hypocone and hypoconal constriction (HC) is also modified significantly through tooth wear. In Early wear I (age 3.5 to 4), the hypocones are slender, HC is obvious. In Early wear II (age 4 to 5), the hypocones are wilder, HC is unapparent. In Early wear III (age 5 to 6), the hypocones are maximal, HC of the premolarsis recognizable, and the molar’s is weak. In Early wear IV (age 6 to 7), all of the hypocones are shorter, HC is weaker. After all, the hypocones stay the same length, HC faded away.
4. The ontogeny of the paracones and metacones can been summarized as follows. Early wear I (age 3.5 to 4), the paracones and metacones of the premolars and M3 are crescent-shaped and are triangular in shape in M1 and M2. In the subsequent stages of Early wear (age 4 to 8), the paracones and metacones of P2 and P3 are elongated trapezoids, varying from a triangle to half round shapes in the remainder of the teeth. To Middle and Late wear, they are square or half round, except for P2. The lingual wall and labial wall of the protoloph and metaloph are stable, with only the space between them increasing from Early wear I to II. The lingual wall of the protoloph bulges angularly in Late wear, and the protoloph changes to a triangular shape. Combining with other known laws of ontogeny, the paracone and metacone may serve as a reliable mark of aging for maxillary cheek teeth.

The ontogenetic laws of maxillary cheek tooth are summarized in the table below (Table 1).

**Table 1:** The main ontogenetic laws of maxillary cheek tooth.

Wear Stage	Opening of posterior wall of the postfossette	Degree of Plications	Protocone	Hypocone	Paracone and Metacone
Early wear I	N	arisen	small spindle	slender, obvious HC	P2/P3/P4/M3 is crescent-shaped, M1/M2 is triangular
Early wear II	N	developed	broaden and round	broaden, weaken HC	P2/P3 is trapezoidal, P4/M1 is triangular, M2/M3 is half-round
Early wear III	P2	immobile	elongate	elongate, weaken HC	P2 is trapezoidal, P3/P4/M1/M2 is triangular, M3 is half-round
Early wear IV	P2 and P3	immobile	immobile	shorten	P2 is trapezoidal, P3 is rectangular P4/M1/M2/M3 is half-round
Early wear V	some of P2	immobile	shorten, smilar to Early wear II	HC faded away	P2 is trapezoidal, P3/P4/M1/M2/M3 is half-round
Middle wear	N	weaken	widen to oval	immobile	P2 is trapezoidal, P3/P4/M1/M2/M3 is half-round or square
Late wear	N	weaken	more wider oval	shorten	P2 is trapezoidal, P3/P4/M1/M2/M3 is square

### Ontogenetic sequences of the mandibular dentitions

The ontogenetic sequences of the mandibular dentitions are listed below. The samples dotted with “*”indicate age reference, and the genders of the samples without labels are unknown.

#### Ontogenetic sequences of the mandibular dentitions of *Hipparion chiai*

**Fig 7:**
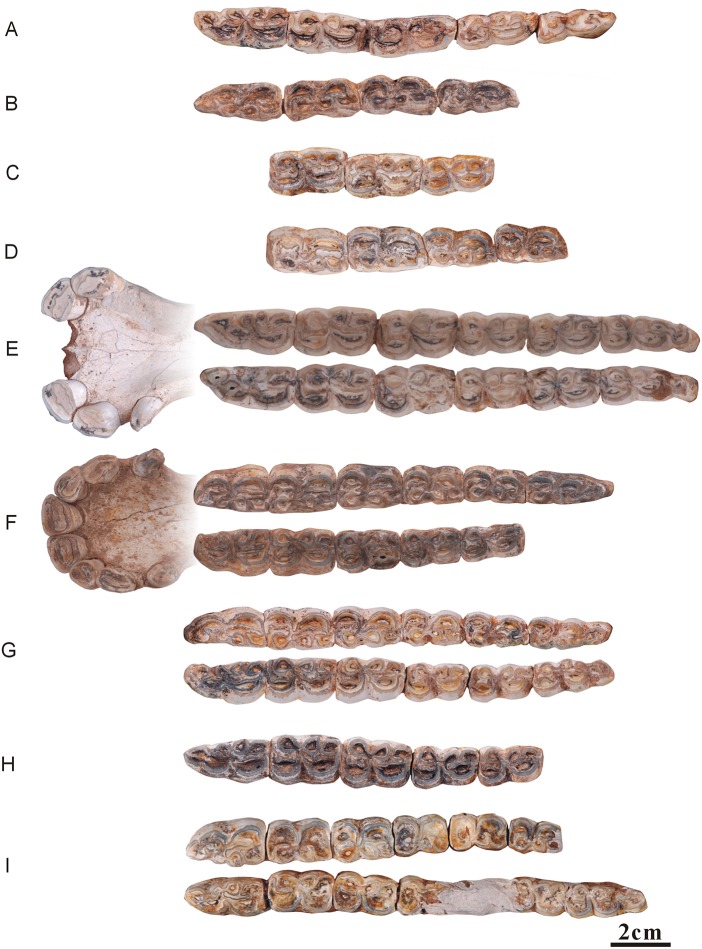
Ontogenetic sequences of the mandibular dentitions of *Hipparion chiai*. (A) NWU1432; (B) NWU1435; (C) NWU1437; (D) NWU1438; (E) NWU1433; (F) NWU1430; (G) NWU1431; (H) NWU1436; (I) NWU1434.

*NWU1432 (Fig 7A): female, juvenile phase (approximately 1.5 years);
NWU1435 (Fig 7B): juvenile phase (approximately 1.5 years);
NWU1437 (Fig 7C): Early wear II (age 4 to 5), the m1’s tooth height is 40.6 mm;
NWU1438 (Fig 7D): Early wear II (approximately 5 years), the m2’s tooth height is 28.8 mm;
*NWU1433 (Figs 3C and 7E): male, Early wear II (approximately 5 years), the average tooth height is 32.4 mm;
*NWU1430 (Figs 3D and 7F): female, Early wear III (age 5 to 6), the average tooth height is 30.4 mm;
NWU1431 (Fig 7G), Early wear III (approximately 5.5 years), the m3’s tooth height is 32.6 mm;
NWU1436 (Figs 3E and 7H), Middle wear (approximately 9 years), the average tooth height is 21.2 mm;
NWU1434 (Fig 7I): Late wear (over 12 years old), the m1 and m2’s tooth height is about 19 mm.
  The measurements are shownin S3 Table.

#### Ontogenetic sequences of the mandibular dentitions of *Hipparion* cf. *coelophyes*

**Fig 8:**
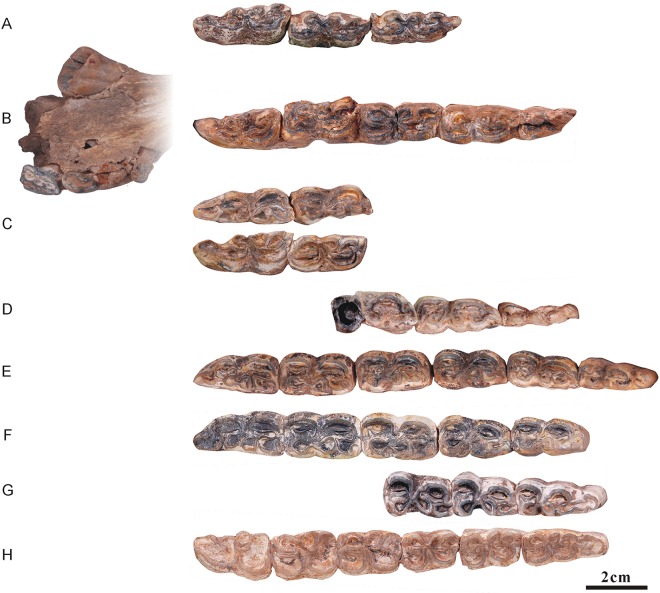
Ontogenetic sequences of the mandibular dentitions of *Hipparion* cf. *coelophyes*. (A) NWU1441; (B) NWU1426.2; (C) NWU1442; (D) NWU1443; (E) NWU1440.1; (F) NWU1439; (G) NWU1444; (H) NWU1445.

*NWU1441 (Fig 8A): Juvenile phase (approximately 5 months);
*NWU1426.2 (Fig 8B), female, juvenile phase (approximately 3/4 year);
NWU1442 (Fig 8C), juvenile phase (less than 1.5 years);
*NWU1443 (Fig 8D), Juvenile phase (age 2.5–3);
*NWU1440.1 (Figs 3A and 8E), Early wear II (age 4–5), the average tooth height is 38.3 mm;
NWU1439 (Figs 3B and 8F), Early wear II (age 4 to 5), the average tooth height is 36.7 mm;
NWU1444 (Figs 3F and 8G), Middle wear (age 8 to 9), the average tooth height for the m1, m2 and m3 is 22.6 mm;
NWU1445 (Figs 3G and 8H), Late wear (over 12 years old), the average tooth height is 11.3 mm.
  The measurements are shown in S4 Table.

#### Analysis of the mandibular dentitions sequences

**Fig 9:**
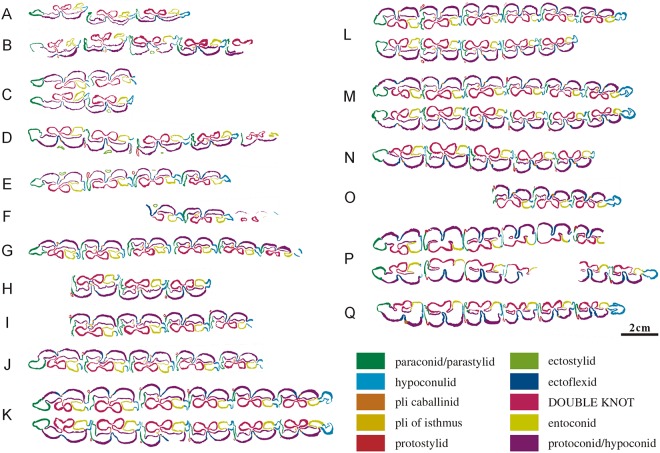
Ontogenetic sequences of mandibular cheek teeth from Lamagou fauna (color line diagram). (A) NWU1441; (B) NWU1426.2; (C) NWU1442; (D) NWU1432; (E) NWU1435; (F) NWU1443; (G) NWU1440.1; (H) NWU1437; (I) NWU1438; (J) NWU1439; (K) NWU1433; (L) NWU1430; (M) NWU1431; (N) NWU1444; (O) NWU1436; (P) NWU1434; (Q) NWU1445.

The morphological differences between *Hipparion chiai* and *H*.cf. *coelophyes* are more ambiguous. Therefore, the analysis is also unclassified and is mainly aimed at the permanent teeth (Fig 9).

1. A sequential opening of the anterior walls of the parastylids in p3 to m3 and the posterior wall of the hypoconulids in p2 to m2 is also clearly discernable, as is the sequence and schedule of the opening in the lower cheek teeth dentition. The opening of the walls in the lower dentition sequence has also been completed by Early wear IV (age 6 to 7).
2. The plications located on the lingual wall of the protoconid and hypoconid are well developed in Early wear II (age 4 to 5), but weaken in Early wear III (age 5 to 6), and remain stable for a long period. The slender plicaballinid is a special morphological discrepancy for the mandibular cheek teeth between *Hipparion chiai* and *Hipparion coelophyes*. The plication located on the anterior wall of the isthmus also displays a tendency from slender in Early wear II (age 4 to 5) to weaker or completely vanished in Early wear IV (age 6 to 7). The protostylids change backward from an encircled ring in Early wear II (age 4 to 5) (starting from p3 and m1) to open on their lingual aspect and elongate labiolingually at late stages.
3. The variation of the isthmuses is obvious in dental ontogeny. The fundamental reason for this is the successive deepening of the ectoflexid due to wear, and the change in premolars is more complicated than the molars. The isthmus of p2 is never divided by the ectoflexid, and the isthmuses of p3 and p4 are incised continually by the ectoflexid from Early wear II (age 4 to 5). Additionally, the postero isthmuses are half formed with the inside end leaning forward. From Early wear III to IV (age 5–7), the postero isthmuses [11] take their form, and the lingual ends of antero and postero isthmuses are linked together in a“∧” shape. In Middle wear and Late wear (after 8 years), the postero isthmus is complete and located parallel to the antero isthmus. The above changes in the antero and postero isthmus of the molars are completed before Early wear IV (age 6 to 7). The linguaflexid becomes narrower downwards, accompanied by the shorten handle and the rounded corners of the double knot. These developments slow before Middle wear (approximately 10 years) but speed up after that.
4. The anterolateral corner of the entoconid becomes rounded gradually, and the connection of the entoconid with the hypoconulid changes wildly at Early wear III (age 5 to 6) and remains stable until Late wear. The hypoconulids of p2 to m2 are compressed by the back tooth and point to lingual at Early wear III (age 5 to 6), then becoming shorter with wear. The paracone and metacone may serve as a reliable mark of aging for mandibular cheek teeth.

The ontogenetic laws of mandibular cheek tooth are summarized in the table below (Table 2).

**Table 2:** The main ontogenetic laws of mandibular cheek tooth.

Wear Stage	Degree of Plications	Protostylid	Depth of Ectoflexid in p3/p4	Depth of Ectoflexid in Molars	Constriction of Entoconid	Hypoconulid
Early wear II	developed	appeared from p3 to m2 in sequence, isolated	half	half	from obvious to weaken in m1/m2	developed
Early wear III	weaken	united in m1	p3 half, p4 more than half	completely	p2 obvious, p3/p4/m1/m2 weaken	developed
Early wear IV	weaken	united in p3	p3 more than half, p4 completely	completely	p2 weaken, p3/p4/m1/m2 gone	squeezed
Early wear V	N	appeared in m3	more than half or completely	completely	N	weaken
Late wear	N	united from p3 to m3	completely	completely	N	gone

## Step sections and analysis

Due to the possible individual differences, the change rules should be confirmed by observing the step section of the isolated hipparionine cheek teeth from Lamagou fauna. The teeth were previously reinforced with transparent resin and were sliced serially with a thickness of approximately 1~3 mm.

### Observation and analysis of maxillary cheek teeth step section

**Fig 10:**
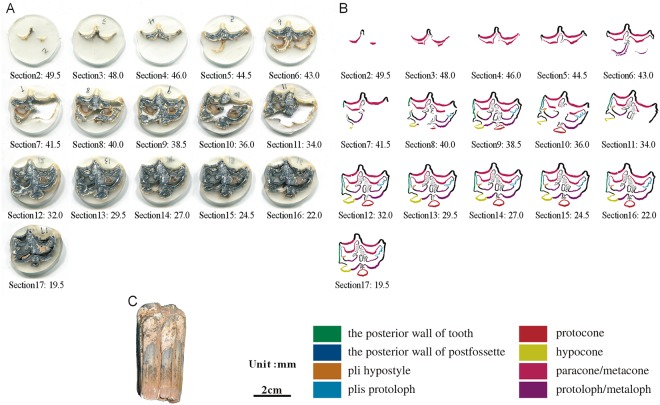
Isolated maxillary tooth NWU1475.7 (P2). (A) photoof step sections of NWU1475.7 (P2); (B) color line diagram of step sections of NWU1475.7 (P2); (C) lateral view of NWU1475.7 (P2).

**Fig 11:**
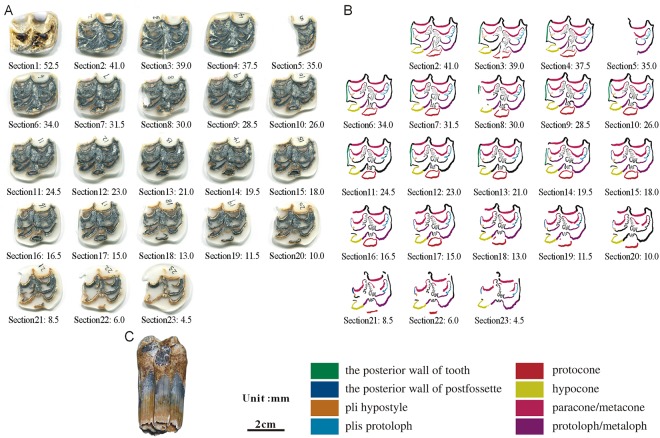
Isolated maxillary tooth NWU1475.8 (P3). (A) photo of step sections of NWU1475.8 (P3); (B) color line diagram of step sections of NWU1475.8 (P3); (C) lateral view of NWU1475.8 (P3).

**Fig 12:**
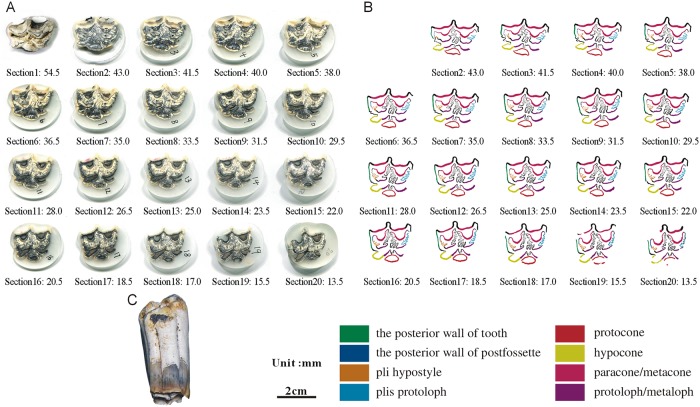
Isolated maxillary tooth NWU1475.9 (M3). (A) photo of step sections of NWU1475.9 (M3); (B) color line diagram of step sections of NWU1475.9 (M3); (C) lateral view of NWU1475.9 (M3).

**Fig 13:**
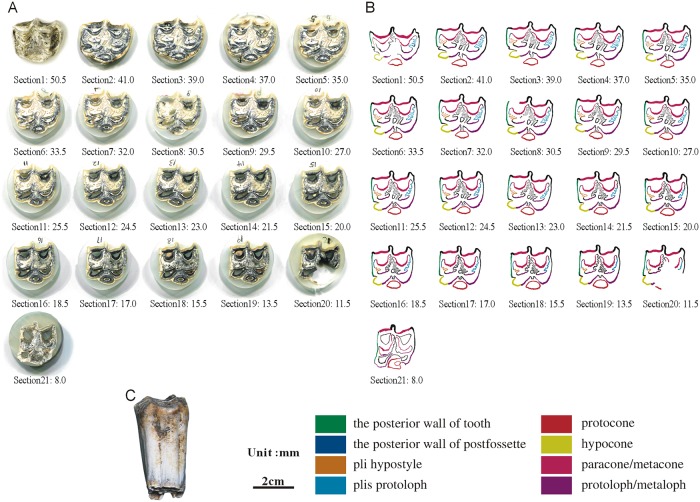
Isolated maxillary tooth NWU1475.10 (M1 or M2). (A) photo of step sections of NWU1475.10 (M1 or M2); (B) color line diagram of step sections of NWU1475.10 (M1 or M2); (C) lateral view of NWU1475.10 (M1 or M2).

Four isolated hipparionine maxillary cheek teeth from the Lamagou fauna were sliced systematically, and their specimen numbers are NWU1475.7 (Fig 10), NWU1475.8 (Fig 11), NWU1475.9 (Fig 12), and NWU1475.10 (Fig 13),of which NWU1475.7-NWU1475.9 are unworn P2, P3, and M3 of *Hipparion chiai* and might originate from the same individual. NWU1475.10 is a mild worn M1 or M2 of *H*. cf. *coelophyes*. The NWU1475.7 was sectioned from top of the cusps. Other samples were sectioned from their widest point to retain the crown. The measurements are shown in S5 Table.

Selected in formation as followed:

1. The posterior walls of the postfossette in NWU1475.7 (Fig 10B), NWU1475.9 (Fig 12B) and NWU1475.10 (Fig 13B) are never “open” in the sections, and in turn, the distance between them and the posterior walls of the teeth remain stable. Only the posterior wall of the postfossette in NWU1475.8 (Fig 11B) is open from Section16 (the height was approximately 16.5 mm) downwards caused by the broken tooth posterior wall.
2. During the process of postfossette closing, the pli hypostyle is located in the lingual wall of the postfossette and points to the labial is shown clearly in Sections 7 to 10 of NWU1475.7 (Fig 10B) and Sections 2 to 4 of NWU1475.9 (Fig 12B). The changes of other maxillary plications conform to the ontogenetic sequences.
3. The protocone changes from punctiform at the apex to spindly (the length-width ratio is 2:1, and both the anterior and posterior end are pointed) in a short period (sections 1 to 11 in NWU1475.7). At the widest section (sections 12 to 14 in NWU1475.7, sections 2 to 10 in NWU1475.8, sections 2 to 7 in NWU1475.9, sections 1 to 6 in NWU1475.10), the length of the protocone is decreasing, whereas the length-width ratio is 3:2, and the anterior end is less pointed. The protocones of the unworn teeth (NWU1475.7~9) are stable for the remainder of the sections, and the width of the protocone increases from sections 16 to 21 in NWU1475.10 (Fig 11B). The change of the protocone from the sections satisfies that from the ontogenetic sequences partly, but the protocone in Late wear is distinctly different from the unworn teeth.
4. The morphological variation of the paracone, metacone, protoloph and metaloph in the sections is generally the same as the ontogenetic sequences. The equivalent relationships among them with the similar occlusal feature are listed as follows: Early wear I is 43 mm and above (above section 6 in NWU1475.7), Early wear II is from 43 to 40 mm (sections7 to 9 in NWU1475.7, section 2 in NWU1475.8, sections 2 to 4 in NWU1475.9, sections 1 to 2 in NWU1475.10), Early wear III is 40 to 36 mm (section 10 in NWU1475.7, sections 3 to 4 in NWU1475.8, sections 5 to 6 in NWU1475.9, sections 3 to 4 in NWU1475.10), Early wear IV is 36 to 32 mm (sections 11 to 12 in NWU1475.7, sections 5 to 6 in NWU1475.8, sections 7 to 8 in NWU1475.9, sections 5 to 7 in NWU1475.10), Early wear V is 32 to 28 mm (section 13 in NWU1475.7, sections 7 to 8 in NWU1475.8, sections 9 to 11 in NWU1475.9, sections 8 to 9 in NWU1475.10), initial Middle wear is 28 to 24 mm (sections 14 to 15 in NWU1475.7, sections 10 to 11 in NWU1475.8, sections 12 to 14 in NWU1475.9, sections 10 to 12 in NWU1475.10), and Late wear is from 17 to 13 mm (sections 17 to 19 in NWU1475.10). It is noteworthy that the shapes are change minimally downward from section 10 in NWU1475.7, section 12 in NWU1475.8, and section 15 in NWU1475.9, and the three teeth show nearly no abrasion. The section height in the equivalent relationships is consistent with the actual teeth height closely.

### Observation and analysis of mandibular cheek teeth step section

**Fig 14:**
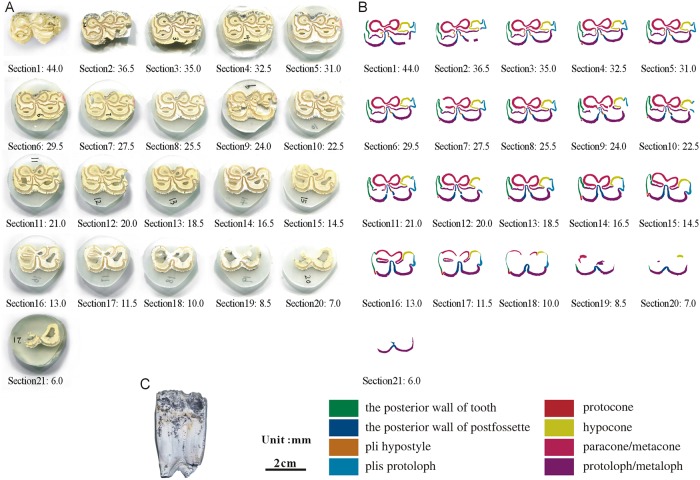
Isolated mandibular tooth NWU1475.11 (p3 or p4). (A) photo of step sections of NWU1475.11 (p3 or p4); (B) color line diagram of step sections of NWU1475.11 (p3 or p4); (C) lateral view of NWU1475.11 (p3 or p4).

**Fig 15:**
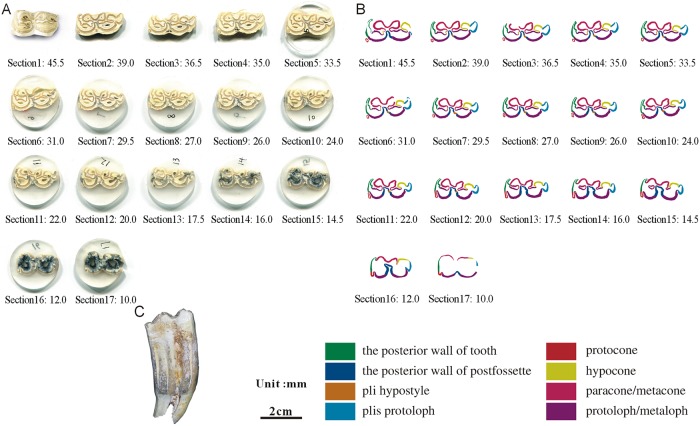
Isolated mandibular tooth NWU1475.12 (m1 or m2). (A) photo of step sections of NWU1475.12 (m1 or m2); (B) color line diagram of step sections of NWU1475.12 (m1 or m2); (C) lateral view of NWU1475.12 (m1 or m2).

Two isolated mildly worn hipparionine mandibular cheek teeth from Lamagou Fauna NWU1475.11 (Fig 14), NWU1475.12 (Fig 15) were sliced systematically, namely, NWU1475.11, a p3 or p4 of *Hipparion* cf. *coelophyes*, and NWU1475.12, an m1 or m2 of *H*. *chiai*. The morphological variation of the mandibular cheek teeth in the step section is consistent with the ontogenetic sequences, although the state of compression cannot be displayed in the step section. The equivalent relationships with the similar occlusal feature are listed as follows: Early wear is 45 to 28 mm (sections 1 to 6 in NWU1475.11, sections 1 to 7 in NWU1475.12); Middle wear is 28 to 18 mm (sections 7 to 13 in NWU1475.11, sections 8 to 12 in NWU1475.12); Late wearis less than 18 mm (below section 14 in NWU1475.11, and section 13 in NWU1475.8). The comparison result is the same as that for maxillary cheek tooth. The measurements are shown in S6 Table.

## Discussion and conclusions

*Hipparion chiai* was initially recognized by Liu et al. [16], and Qiu et al. [5] identified its validity and placed it in the subgenus *Hippotherium* of *Hipparion*. Subsequently, the occurrence of *H*. *chiai* or *H*. cf. *chiai* was successively reported from the Lamagou fauna of Fugu, Shaanxi [12–14], the Shengou fauna of Qaidam Basin [17], the Dashengou fauna in the Liushu group of Linxia Basin in Gansu [18], and Qinan in Gansu [19]. Liu [20] studied new materials of *H*. *chiai* collected from Lantian, Shaanxi in terms of morphology and phylogeny, and treated *Hippotherium* as a genus for classic taxonomic, which is consistent with the mainstream views in the west [21–24]. However, for consistency and completeness of the regional researches, *Hippotherium* remains a subgenus as the previous literatures [5, 12–14, 16–18] in this study. The stratigraphic range of *H*. *chiai* in Shaanxi, Gansu, Nei Mongol of northern China were characterized as middle to late Bahean (ca. 9.9–7 Ma) [12–14, 17–19, 25–28]. *H*. *chiai* is characterized by the following features: medium size; single and sub-rhomboid preorbital fossa (PF); well- defined anterior rim of the PF; well developed posterior pocket of the PF; PF located far from the orbit (>40 mm); shallow nasal slit with a posterior edge located anterior to P2; low-crowned teeth (medial cheek tooth height is 50 to 55 mm); slender anterostyle with grooves on both lateral; elongated protocone with weak anterolateral thorn; simple enamel plication; double plicaballin; pli hypostyle located on the interior wall of postfossette and points lateral; weak hypoconal constriction; angular metastylid; v-shaped linguaflexid; wide and low ectoflexid; exist entprotostylid; weak plicabalinid [5, 16, 20].

The protocone, hypocone and plis in fossea are important recognition features of hipparionine horses, the morphological changes in wear present many puzzles for identification and comparison. The ontogenetic sequence can also establish a framework for more accurate and valid intraspecific or interspecific comparison. Using this framework, the following description can be noted: wilder anterostyle with deeper lingual groove, shorter protocone and hypocone, the pli hypostyle of the premolars located further back, deeper ectoflexid, and rounder metastylid in the *Hipparion chiai* from the Lamagou fauna, Fugu [12–14] in contrast with that from the Lantian, Shaanxi [16] and the Shengou fauna of the Qaidam Basin [17] at the same age. Therefore, the *H*. *chiai*from the Lamagou fauna, Fugu [12–14] is *H*. cf. *chiai* exactly, and the detailed description, comparison (in preparation) and further phylogenetics (under study) will be published in another papers. Certain new clues of phylogeny might arise for the different geological ages.

The other hipparionine species in the Lamagou fauna is *Hipparion* cf. *coelophyes*, but was mistaken as *H*. cf. *fossatum* [12–14]. *H*. *coelophyes* and *H*. *fossatum* were initially recognized by Sefve [29], Qiu et al. [5] considered that *H*. *coelophyes*is valid (the revision in the footnote), but *H*. *fossatum* was used in the main text. Besides, *H*. *fossatum* was revised by Forstén [30], Watable [31] grouped *H*. *forstenae* into the *H*. *fossatum*revised by Forstén [26], and Zouhri and Bensalmia [21] indicated that *H*. *fossatum*is the synonym of *Cremohipparion forstenae*. The *H*. *fossatum* lacks consensus and is assigned different identifications in different references.

The *Hipparion* cf. *coelophyes* from the Lamagou fauna was identified [12–14] following the retrieval by Qiu et al. [5]. The characteristics are listed as follows: single preorbital fossaare located moderately far from the orbit; the nasal slit is located above P2; medium-sized, moderate preorbital fossa without a ridged anterior rim; disunited hypocone; the plications in pre- and postfossettes are complex; the protocone is oblate and spindle-shaped; only the metastylid is angular; and a thin placation is located on the anterior wall of the hypocind and points forward [5, 32]. Therefore, the size and locations of the preorbital fossa and nasal slit, the hypocone and the plications of *H*. *coelophyes* are the same as those of *H*. *chiai*. The development and anterior rim of the preorbital fossa might have become distorted by geological processes; furthermore, the oblate and spindle-shaped protocone and the thin placation on the anterior wall of the hypocind were proved to be temporary in Early wear II and V by the ontogenetic sequence. Thus, it can be observed that the *H*. cf. *coelophyes* of the Lamagou fauna is still *H*. cf. *chiai*.

After studying the ontogenetic sequences and the step sections, the variability of tooth wear in *Hipparion* cf. *chiai* of the late Miocene in northern China was well documented and is expected to form the basis for quantitative paleontology by CG (computer graphics) and serve as a good example and comparable information for another hipparionine genus-species. However, the hipparionine species of the Lamagou fauna are low crowned (HI is approximately 4.4). Thus, the feature of the crown in each age would be highly different from that of Pliocene hypsodont hipparionine. The current study was aimed at introducing an observation method for morphology of hipparionine cheek tooth, and we hope it can be replicated to the following specific works:

1. This will help to clarify the intraspecies variation and reduce the classification error caused by aging.
2. Enabling the truly species differences, particularly those that between the similar species. Those differences may significantly indicate the evolutionary polarity and the direction, however, the variables of age make it much harder for researchers to notice these differences.
3. Quantitative analysis for morphology by computer graphics, which need the appropriate comparative models to reach the more reasonable results. The framework which was established by ontogenesis sequence would be an appropriate comparative model for calculating the evolutionary rate and/or intelligent identification.

This work might greatly promote the morphological and taxonomic study of hipparionines.

## Supporting information

S1 Table Measurement of the maxillary cheek teeth of *Hipparion chiai* in dentitions (mm). Maxillary cheek tooth measurements: M1. tooth height; M2. occlusal length; M3. occlusal length of the protocone; M4. occlusal breadth; PF. plication formula [11].
(DOCX)

S2 Table Measurement of the maxillary cheek teeth of *Hipparion* cf. *coelophys* in dentitions (mm). Maxillary cheek tooth measurements: M1. tooth height; M2. occlusal length; M3. occlusal length of the protocone; M4. occlusal breadth; PF. plication formula [11].
(DOCX)

S3 Table Measurement of the mandibular cheek teeth of *Hipparion chiai* in dentitions (mm). Mandibular cheek tooth measurements: M1. tooth height; M2. tooth length; M3. length of the preflexid; M4. length of the double knot; M5. length of the postflexid; M6. occlusal breadth.
(DOCX)

S4 Table Measurement of the mandibular cheek teeth of *Hipparion* cf. *coelophyes* in dentitions (mm). Mandibular cheek tooth measurements: M1. tooth height; M2. tooth length; M3. length of the preflexid; M4. length of the double knot; M5. length of the postflexid; M6. occlusal breadth.
(DOCX)

S5 Table Measurement of sectioned maxillary teeth (mm). Maxillary cheek tooth measurements: M2. occlusal length; M3. occlusal length of the protocone; M4. occlusal breadth; PF. plication formula [11].
(DOCX)

S6 Table Measurement of sectioned mandibular teeth (mm). Mandibular cheek tooth measurements: M2. tooth length; M3. length of the preflexid; M4. length of the double knot; M5. length of the postflexid; M6. occlusal breadth.
(DOCX)

## References

[1] Gidley JW. Tooth characters and revision of the North American species of the North American species of genus *Equus*. Bull. Amer. Mus. Nat. Hist. 1901; 14:91–141.

[2] Teryaev VA. Paleontological determination of age of vertebrates. Problemy Paleontologii. 1936; 1:135–169.

[3] Wehrli H. Beitrag zur Kenntnis der ‘Hipparionen’ von samos. *Pal*. *Zettschr*. 1941; 22:321–386.

[4] Gromova VI. Gippariony (rod Hipparion) pomaterialam Taraklii, Pavlodara in drugim. Trudy Paleont. 1st ed. AKad: Nauk SSSR; 1952.

[5] Qiu ZX, Huang WL, Guo ZH. The Chinese hipparionine fossils. *Palaeont Sin New Ser C*; 1987.

[6] Pang LB. Fossils of Pliocene Hipparion (Equidae, Perissodactyla) from Gaotege locality, Nei Mongol and their implications. Vertebrata PalAsiatica. 2011; 49:210–222.

[7] Bernor BL, Sun BY. Morphology through ontogeny of Chinese *Proboscidipparion* and *Plesiohipparion* and observations on their Eurasian and African relatives. Vertebrata PalAsiatica. 2015; 53:73–92.

[8] Dai T, Zhang XL. Ontogeny of the eodiscoid trilobite Tsunyidiscus acutus from the lower Cambrian of South China. Palaeontology. 2011; 54:1279–1288.

[9] Dai T, Zhang XL. Ontogeny of the redlichiid trilobite Metaredlichia cylindrical from the lower Cambrian (Cambrian Stage 3) of South China. Journal of Paleontology. 2012; 86:647–652.

[10] Dai T, Zhang XL. Ontogeny of the redlichiid trilobite Eoredlichia intermedia from the Chengjiang Lagerstätte, lower Cambrian, southwest China. Lethaia. 2013; 46:262–273.

[11] Eisenmann V, Alberdi TM, Giuli C, Staesche U. Studying fossil horses, volume: methodology. Leiden: E.J. Brill; 1988.

[12] Xue XX, Zhang YX, Yue LP. The discovery of *Hipparion* fauna of Laogaochuan and its division of eras, Fugu County, Shaanxi. Chinese Science Bulletin. 1995; 40:447–449.

[13] Zhang YX, Xue XX, and Yue LP. Age and division of Neogene “Red Bed” of Laogaochuan, Fugu County, Shaanxi. Journal of Stratigraphy. 1995; 19:214–219.

[14] Zhang YX, Xue XX, Yue LP. Assemblages of *Hipprion* Fauna in Northern Shaanxi. Journal of Northwest University (Natural Science Edition). 1995; 25:483–486.

[15] Sisson S. The anatomy of the domestic animals. Philadelphia, PA: W.B. Saunders Company; 1953.

[16] Liu DS, Li CK, Zhai RJ. Pliocene verterbrates of Lantian, Shensi. Profes Papers of Stratigraphy and Palaeontology. 1978; 7:149–200.

[17] Deng T, Wang XM. Late Miocene Hipparion (Equidate, Mammalia) of eastern Qaidam Basin in Qinghai, China. Vertebrata PalAsiatica. 2004; 42:316–333.

[18] Deng T, Qiu ZX, Wang BY, Wang XM, Hou SK. Late Cenozoic biostratigraphy of the Linxia Basin, northwestern China In: Wang XM, editors. Fortelius M. Fossil Mammals of Asia. Neogene Biostratigraphy and Chronology. New York: Columbia University Press; 2013pp. 243–273.

[19] Zhang ZQ, Kaakinen A, Liu LP, Sen S, Grose AW, Qiu ZD, et al Mammalian Biochronology of the Late Miocene Bahe Formation In Wang XM, editors. Fortelius M. Fossil Mammals of Asia. Neogene Biostratigraphy and Chronology. New York: Columbia University Press; 2013pp. 187–202.

[20] Liu Y. Late Miocene Hipparionine fossils from Lantian, Shaanxi Province and phylogenetic analysis on Chinese Hipparionines. Ph. D dissertation, University of Chinese Academy of Sciences.2013.

[21] Zouhri S, Bensalmia A. Révisionsystématique *des Hipparionsensu* lato (Perissodactyla, Equidae) de l’Ancien Monde. Estudios Geologicos. 2005; 61:61–99.

[22] Bernor RL, Koufos GD, Woodbune MO, Fortelius M. The Evolutionary History and Biochronology of European and Southwest Asian Late Miocene and Pliocene Hipparionine horses In: Bernor BL, Fahlbusch V, Mittmann HW, editors. The Evolution of Western Eurasian Neogene Mammal Faunas. New York: Columbia University Press; 1996 pp. 307–338.

[23] Bernor RL, Kaiser TM, Nelson SV, Rook L. Systematics and Paleobiology of Hippotherium malpassi n. sp. from the latest Miocene of Baccinello V3 (Tuscany, Italy). Bolletino della Societa Paleontologica Italiana.2011; 50: 175–208.

[24] Bernor RL, Ataabadi MM, Meshida K, Wolf D. The Maragheh Hipparions; Late Miocene of Azerbaijan, Iran. Palaeobiology and Palaeodiversity. 2016; 96: 453–488.

[25] Kaakinen A. A long terrestrial sequence in Lantian—a window into the late Neogene palaeoenvironments of northern China. Helisinki: Publications of the Department of Geology D4; 2005.

[26] Fang XM., Zhang WL, Meng QQ, Gao JP, Wang XM, King J, et al High-resolution magnetostratigraphy of the Neogene Huaitoutala section in the eastern Qaidam Basin on the NE Tibetan Plateau, Qinghai Province, China and its implication on tectonic uplift of the NE Tibetan Plateau. Earth and Planetary Science Letters. 2007; 258:293–306.

[27] Wang XM., Qiu ZD, Li Q, Wang BY, Qiu ZX, Downs RW, et al Vertebrate paleontology, biostratigraphy, geochronology, and paleonenvironment of Qaidam Basin in northern Tibetan Plateau. Palaeogeography, Palaeoclimatology, Palaeoecology. 2007; 254:363–385.

[28] Zhang J, Xie GP, Li JJ, Song CH, Zhao ZJ, Wang XX, et al Mammal fossils and ecological environment features of the Neogene from Qin’an area, Gansu province. Quaternary Sciences. 2011; 31:614–621.

[29] Sefve I. Die Hipparionen Nord-Chinas. Palaeontologia Sinica. Series C. 1927; 4(2):1–93.

[30] Forstén AM. Chinese Turolian Hipparion in the Lagrelius Collection In: Lucas SG, Matter NJ, editors. Studies in Chinese fossil vertebrates. New Series: Bulletin of the Geological Institution of the University of Uppsala; 1985 pp. 113–124.

[31] Watable M. Phylogenetic relationship between Chinese and Western Old World Hipparionines (Equidae, Perissodactyla). Humans and Nature. 1992; 1:35–36.

[32] Pang LB. Fossils of hipparionin Shilei locality, Linxia Basin. M. A. thesis, University of Chinese Academy of Sciences.2010.

