# Supplementary material for: Assessment of dental ontogeny in late Miocene hipparionines from the Lamagou fauna of Fugu, Shaanxi Province, China
Source: PLoS One. 2017 Apr 26;12(4):e0175460. doi: 10.1371/journal.pone.0175460 (PMC5405952; doi:10.1371/journal.pone.0175460)
Supplement: S3 Table — Mandibular cheek tooth measurements: M1. tooth height; M2. tooth length; M3. length of the preflexid; M4. length of the double knot; M5. length of the postflexid; M6. occlusal breadth. (DOCX) [file pone.0175460.s003.docx]

**S3 Table.**
