# Supplementary material for: Assessment of dental ontogeny in late Miocene hipparionines from the Lamagou fauna of Fugu, Shaanxi Province, China
Source: PLoS One. 2017 Apr 26;12(4):e0175460. doi: 10.1371/journal.pone.0175460 (PMC5405952; doi:10.1371/journal.pone.0175460)
Supplement: S5 Table — Maxillary cheek tooth measurements: M2. occlusal length; M3. occlusal length of the protocone; M4. occlusal breadth; PF. plication formula [11]. (DOCX) [file pone.0175460.s005.docx]

**S5 Table.**
